# Long-term live-cell lipid droplet-targeted biosensor development for nanoscopic tracking of lipid droplet-mitochondria contact sites

**DOI:** 10.7150/thno.59848

**Published:** 2021-06-22

**Authors:** Chengying Zhang, Huarong Shao, Jie Zhang, Xinyan Guo, Yue Liu, Zhigang Song, Fei Liu, Peixue Ling, Longguang Tang, Kang-Nan Wang, Qixin Chen

**Affiliations:** 1 School of Pharmaceutical Sciences, Shandong University, Jinan 250101, PR China.; 2 Institute of Materia Medica, Shandong First Medical University & Shandong Academy of Medical Sciences, Jinan 250062, PR China.; 3 Shunde Hospital of Southern Medical University (The First People's Hospital of Shunde), Foshan, Guangdong 528308, PR China.; 4 International Institutes of Medicine, The 4th Affiliated Hospital of Zhejiang University School of Medicine, Yiwu, Zhejiang 322000, China.; 5 Shandong Academy of Pharmaceutical Science, Key Laboratory of Biopharmaceuticals, Engineering Laboratory of Polysaccharide Drugs, National-Local Joint Engineering Laboratory of Polysaccharide Drugs, Jinan 250101, PR China.; 6 Advanced Medical Research Institute/Translational Medicine Core Facility of Advanced Medical Research Institute, Shandong University. Jinan 250101, PR China.; 7 College of Basic Medicine, Jining Medical University, Jining 272067, PR China.

**Keywords:** contact sites, lipid droplets, mitochondria, extended-resolution imaging

## Abstract

**Background:** Lipid droplets (LDs) establish a considerable number of contact sites with mitochondria to enable energy transfer and communication. In this study, we developed a fluorescent biosensor to image LD-mitochondria interactions at the nanoscale and further explored the function of LD-mediated matrix transmission in processes involving multi-organelle interactions.

**Methods:** A fluorescent probe called **C-Py** (C_21_H_19_N_3_O_2_, 7-(diethylamino) coumarin-3-vinyl-4-pyridine acetonitrile) was designed and synthesized. Colocalization of **C-Py** and the commercial LD stain Nile Red was analyzed in HeLa cells. The fluorescence stability and signal to background ratio of **C-Py** under structured illumination microscopy (SIM) were compared to those of the commercial probe BODIPY493/503. The cytotoxicity of **C-Py** was assessed using CCK-8 assays. The uptake pattern of **C-Py** in HeLa cells was then observed under various temperatures, metabolic levels, and endocytosis levels. Contact sites between LDs and various organelles, such as mitochondria, nuclei, and cell membrane, were imaged and quantitated using SIM. Physical changes to the contact sites between LDs and mitochondria were monitored after lipopolysaccharide induction.

**Results:** A LD-targeted fluorescent biosensor, **C-Py**, with good specificity, low background signal, excellent photostability, low cytotoxicity, and high cellular permeability was developed for tracking LD contact sites with multiple organelles using SIM. Using **C-Py**, the subcellular distribution and dynamic processes of LDs in living cells were observed under SIM. The formation of contact sites between LDs and multiple organelles was visualized at a resolution below ~200 nm. The number of LD-mitochondria contact sites formed was decreased by lipopolysaccharide treatment inducing an inflammatory environment.

**Conclusions: C-Py** provides strategies for the design of ultra-highly selective biosensors and a new tool for investigating the role and regulation of LDs in living cells at the nanoscale.

## Introduction

Lipid droplets (LDs) are important structures in eukaryotic cells that are not only responsible for energy storage and supply 1, 2 but also play essential roles in the maintenance of cell homeostasis, including organelle energy balance, and confer protection against lipotoxicity and apoptosis. LDs also demonstrate intimate interactions with other intracellular organelles 3-5. LDs can move along the cytoskeleton and interact with various subcellular organelles, such as mitochondria and nuclei, to regulate intracellular signal transduction and metabolic homeostasis in physiological and pathological environments 6-9. LDs and mitochondria have been shown to play central roles in inflammatory and infectious conditions. Prior evidence has demonstrated that inflammation stimulates the assembly of multiple host defense proteins into complex clusters on LDs, promoting physical and functional uncoupling of LDs from mitochondria, which indicates that inflammation reduces fatty acid metabolism 10. Further, it has been shown that LDs actively participate in the development of innate immunity in mammals. Although it is of considerable significance to understand the interactions between mitochondria and LDs to develop therapeutic strategies for treating immune-related diseases, no studies thus far have monitored the interactions between these two intracellular organelles under inflammatory and infectious conditions. Therefore, the mechanisms by which LDs and mitochondria behave under inflammatory and infectious conditions remain elusive.

Fluorescence microscopy is a powerful tool for studying in detail the interaction networks between subcellular organelles. However, the nanoscale morphological changes that LDs and organelles experience under *in vivo* inflammatory and infectious conditions cannot be visualized using conventional morphological evaluation methods since it remains difficult to distinguish subcellular structures smaller than 200 nm 11-16, which hinders research aimed at elucidating the underlying mechanisms. Recently developed extended-resolution microscopy techniques, including stimulated emission depletion (STED), stochastic optical reconstruction microscopy (STORM), photoactivated localization microscopy (PALM), and structured illumination microscopy (SIM), have enabled visualization of molecular interactions at the nanoscale in living cells 17-20. However, usage of commercial LD dyes (e.g., Nile Red, BODIPY 493/503) presents with issues such as high background noise and poor photostability 21-24. In addition, contact is a dynamic process in which organelle and organelle are approached, contacted, and then separated 14, 25. Therefore, visualization of LD biogenesis and contact sites established with other organelles (such as mitochondria) at the nanoscale remains a serious challenge. Thus, development of a novel biosensor compatible with extended-resolution imaging is necessary for the investigation of diagnosis and treatment strategies for LD-related diseases.

Herein, a LD-targeted biosensor, C_21_H_19_N_3_O_2_, (*Z*)-3-(7-(diethylamino)-2-oxo-2*H*-chromen-3-yl)-2-(pyridin-4-yl) acrylonitrile, **C-Py**, was developed to trace the interactions of LDs with other organelles and the behavior of LDs under inflammatory and infectious conditions using SIM. Owing to its excellent photostability, low toxicity, good cell permeability, and high signal to background ratio, **C-Py** was successfully used for SIM of LDs to detect their quantity, morphological changes, and dynamic processes in living cells. SIM revealed that LDs establish physical contacts with a variety of organelles, such as mitochondria, nuclei, and cell membrane, and that part of the distance between LDs and mitochondria contact sites is shorter than ~200 nm. Physical separation of LDs and mitochondria after lipopolysaccharide (LPS) induction in living cells was also observed at the nanoscale. **C-Py** may be used as a biosensor for tracking LD dynamics under SIM, providing a powerful approach for investigating diagnosis and treatment strategies for LD-related diseases.

## Materials and methods

### General materials

Dulbecco's modified Eagle's medium (#11965118, DMEM), phenol-free medium (#1894117), Penicillin-streptomycin (#15140163, 10,000 units/ml), Trypsin-EDTA (#25200-072) and other reagents for cell culture were obtained from Gibco BRL (Grand Island, NY, USA). Fetal bovine serum (FBS) was obtained from VivaCell Shanghai (Shanghai, China). Lipopolysaccharide (#L2630, LPS) was obtained from Sigma-Aldrich (WGK, Germany). Nile Red (#N1142), MitoTracker^®^ Deep Red FM (#M22426, MTDR), 4',6-Diamidino-2-Phenylindole, Dihydrochloride (#D1306, DAPI), (1,1'-Dioctadecyl-3,3,3',3'-Tetramethylindocarbocyanine Perchlorate ((#D3911, DiL) were obtained from Invitrogen (Eugene, Oregon, USA). HeLa cells, Hep G2 cells and A549 cells were gifted from Fengshan Wang's lab (Shandong University).

### Synthesis and characterization

7-N, N diethylamino-coumarin-3-carbaldehyde (3) in **Scheme S1** was synthesized according to literature method 26. A mixture of (3) (0.180 g, 0.73 mmol) and 4-pyridineacetonitrile (0.087 g, 0.73 mmol) were placed in a 50 mL of flask with 20 mL dry ethanol for 24 h, and the crude product of **C-Py** was obtained. After cooling to room temperature, the crude product was filtered, and washed with cool acetonitrile. Finally, high purity **C-Py** was obtained. ^1^H NMR (400 MHz, CDCl_3_) δ 8.82 (s, 1H), 8.66 (d, *J* = 4.6, 1.6 Hz, 2H), 8.10 (s, 1H), 7.56 (dd, *J* = 4.6, 1.7 Hz, 2H), 7.43 (d, *J* = 9.0 Hz, 1H), 6.65 (d, *J* = 9.0, 2.5 Hz, 1H), 6.50 (d, *J* = 2.3 Hz, 1H), 3.48 (q, *J* = 7.1 Hz, 4H), 1.26 (t, *J* = 7.1 Hz, 6H). ^13^C NMR (101 MHz, CDCl3) δ 161.57 (s), 157.17 (s), 152.68 (s), 150.50 (s), 142.09 (s), 141.75 (s), 137.97 (s), 131.30 (s), 119.58 (s), 117.48 (s), 112.47 (s), 110.06 (s), 108.50 (s), 106.13 (s), 97.13 (s), 77.43 (s), 77.11 (s), 76.79 (s), 45.20 (s), 12.50 (s). HRMS (positive mode): For [M+H] ^+^ m/z 346.16. Found: [M+H] ^+^ m/z 346.20.

### Cell culture and imaging under OMX 3D-SIM

HeLa cells at a density of 1×10^5^ were seeded on 35 mm glass-bottom culture dishes and incubated with 2 mL of DMEM medium supplemented with 10% FBS. After 24 h incubation, cells were incubated with 10 μM **C-Py** for 2 h, and washed with fresh DMEM for five times. Lastly, the cells were cultured in a phenol-free medium and imaged under an OMX 3D-SIM extended-resolution microscope (Delta Vision, Inc) equipped with an 60×/1.42 numerical aperture oil-immersion objective lens and solid-state lasers 27. Images were obtained at 512 × 512 using Z-stacks with a step size of 0.125 μm, and **C-Py** was excited at 488 nm and emitted at 505-550 nm.

### Cytotoxicity assay

The cytotoxicity assay was measured with the Cell Counting Kit-8 (CCK-8) assay. HeLa cells were seeded in seven 96-well plates at a density of 5×10^3^ cells/well in DMEM with 10% FBS at 37 °C for 24 h. Then the medium culture was replaced with 1000 μL fresh medium containing 0, 0.1, 0.5, 1.0, 5.0, 10, 50 μM of **C-Py**. After 1, 3, 6, 12 h, respectively, 10 μL CCK8 solution was added to each well, and the plate was incubated in the incubator for 1 h. Finally, the absorbance at 450 nm was determined by enzyme-linked immunosorbent assay.

### Flow cytometry analysis

HeLa cells were seeded in 6-well plates at a density of 1×10^5^ cells/well in DMEM with 10% FBS at 37 °C for 24 h. Then the medium culture was replaced with 1000 μL fresh medium containing 0, 0.1, 0.5, 1.0, 2.5, 5.0, 10, 25, 50 μM of **C-Py**. After being treated with biosensors for 2 h, the cells were digested by trypsin and washed twice with precooled PBS. Cells were resuspended with 500 μL buffer and detected by flow cytometry. A total of 10,000 cells per sample were collected with a BD Accuri C6^®^ flow cytometer (BD Biosciences, San Jose, CA, USA), and the mean fluorescence intensity detected by FL1-H (533 ± 30 nm) were analyzed using FlowJo software (Becton Dickinson, Sparks, MD, USA).

### Co-localization experiments

Cells at a density of 1×10^5^ were seeded on 35 mm glass-bottom culture dishes and incubated with 2 mL of DMEM medium supplemented with 10% FBS. After 24 h incubation, cells were incubated with 10 μg/mL Nile Red or 100 nM MTDR for 30 min, and 1000 ng/mL DAPI for 5 min. Then cells washed with fresh DMEM three times and incubated with 10 μM **C-Py** for 2 h. Finally, the cells were cultured in a phenol-free medium and imaged under an OMX 3D-SIM. Nile Red was excited at 561 nm and emissed at 575 nm, MTDR at 647 nm and emissed at 663-735 nm, while excitation wavelength of DAPI was 364 nm and emission at band of 454 nm. The images were analyzed using ImageJ.

### Confocal laser scanning microscopy

HeLa cells adhered to 35 mm glass-bottom culture dishes were incubated with 10 μM **C-Py** in DMEM for 2 h at 37 °C. Then samples were analyzed under a laser scanning microscope LSM 710 META (Carl Zeiss, Jena, Germany) equipped with 63×/1.49 numerical aperture oil immersion objective lens. Images were acquired with ImageJ software (version 1.51j8, National Institutes of Health) and ZEN software (version 2012 SP1) (Carl Zeiss, Inc.).

### Electron microscopy

Cells were seeded on glass-bottom culture dishes and incubated with DMEM medium supplemented with 10% FBS. After 24 h, cells were collected by centrifugation at 1,000 g and put into 2.5% glutaraldehyde, fixed with 1% osmium acid. Then the mixture was dehydrated and embedded in conventional ethanol, ultrathin section, double staining with sodium acetate and lead citrate, and finally observed by transmission electron microscope (JEM 100CX II transmission electron microscope, Tokyo, Japan) with an acceleration voltage of 80 kV.

## Results and Discussion

### Biosensor design and characterization

**Figure 1:**
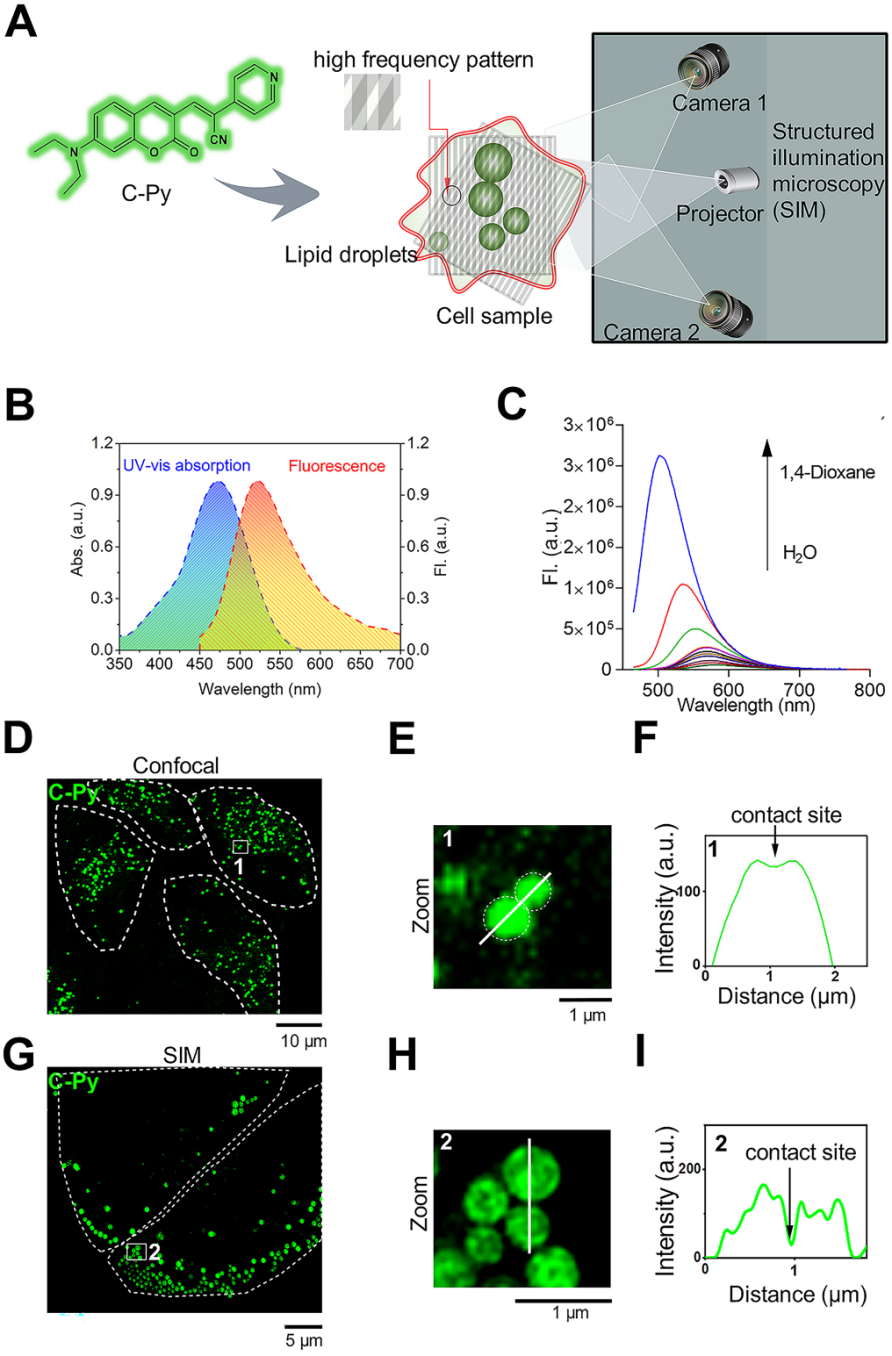
Design and optical characterization of the biosensor **C-Py**. (A) Schematic representation of SIM of LDs labelled with **C-Py**. (B) UV-vis absorption and emission spectra of **C-Py** (10 μM) in buffer solution (10 mM Tris-HCl, 100 mM KCl, pH 7.4). (C) Fluorescence emission spectra of **C-Py** in mixtures of 1,4-dioxane and water with various proportions (*λ*_ex_ = 405 nm). (D) Confocal microscopy image of **C-Py**-labeled particles. (E) Enlarged image of the indicated region in D. (F) Fluorescence intensity profile along the line drawn in E across a contact site between **C-Py**-labeled particles. (G) SIM image of **C-Py**-labeled particles. (H) Enlarged image of the indicated region in G. (I) Fluorescence intensity profile along the line drawn in H across a contact site between **C-Py**-labeled particles.

LDs are highly complex and dynamic intracellular structures that store neutral lipids, including triglycerides and cholesterol esters, within cells and possess a phospholipid monolayer covered with various proteins 1, 2. Considering the structural characteristics of LDs, biosensors equipped with suitable lipophilic units may efficiently target LDs. The 7-*N*, *N*-diethylamino coumarin unit, with a hydrophobic structure similar to that of Nile Red (**Figure S1**), has frequently been used in the design of LD biosensors 28. Donor-π-acceptor (D-π-A) fluorescent materials may present rich photophysical properties. Modification of the structures of the donors and acceptors can enable design and optimization of biosensors with desired excitation and emission wavelengths 29, 30. Thus, **C-Py** was designed to contain a coumarin group and a pyridine unit to form a D-π-A structure, which not only satisfied the required photophysical properties but also resulted in a biosensor with the desired hydrophilicity/lipophilicity for uptake by living cells (**Figure 1A**). The synthesis route and characterizations (HR-MS, ^1^H NMR, and ^13^C NMR) of **C-Py** are illustrated in **Scheme S1** and **Figures S2-S4**, which provide evidence for the structure and high purity of the compound.

The UV-vis absorption and emission spectra of **C-Py** are shown in **Figure 1B**. **C-Py** showed an absorption peak at 476 nm (*ε* = 2.5×10^4^ mol^-1^ cm^-1^) and a fluorescence peak at 561 nm. It was weakly emissive in pure water, but its fluorescence gradually increased as the solvent polarity decreased. As shown in **Figure 1C**, with increasing 1,4-dioxane content, the emission intensity of **C-Py** gradually increased, accompanied by an apparent blue-shift in the maximum emission from 561 nm to 503 nm due to intramolecular charge transfer 31. The high lipophilicity of **C-Py** and its strong fluorescence emission in low-polarity solvents demonstrate its suitability for applications in LD imaging.

To examine the ability of **C-Py** to visualize contact sites between organelles, confocal microscopy (**Figure 1D**) and SIM (**Figure 1G**) were used to observe contact sites between **C-Py**-labeled particles. As expected, the edges of the particles could not be clearly observed using conventional confocal microscopy (**Figure 1E**), and data on the contact sites between particles could not be extracted (**Figure 1F**). In contrast, contact sites between **C-Py**-labeled particles could be resolved under SIM (**Figure 1H-I**). These results suggest that **C-Py** could be used to observe contact site events occurring in living cells under SIM. In addition, bright-field images of **C-Py**-labeled particles collected by confocal microscopy and SIM were clear (**Figure S5**), which also demonstrates that SIM has high imaging resolution. In order to assess the influence of immersion oil on SIM image quality, we compared immersion oils with refractive indices (*n*@589.3 nm) of 1.516, 1.524, and 1.510. The reconstructed images in **Figure S6A-C** were imaged identically but were acquired with the three different immersion oils. The image collected with the optimal immersion oil (*n* = 1.516, A) is clearer than those collected with the other oils. The widefield images in **Figure S7A-C** were similarly acquired with identical parameters but different oils. As can be seen from the signal-to-background ratios (SBR) displayed for each channel, imaging with an incorrect oil leads to significantly decreased SBR compared with the optimal oil.

### Extended-resolution imaging of C-Py in living cells

**Figure 2:**
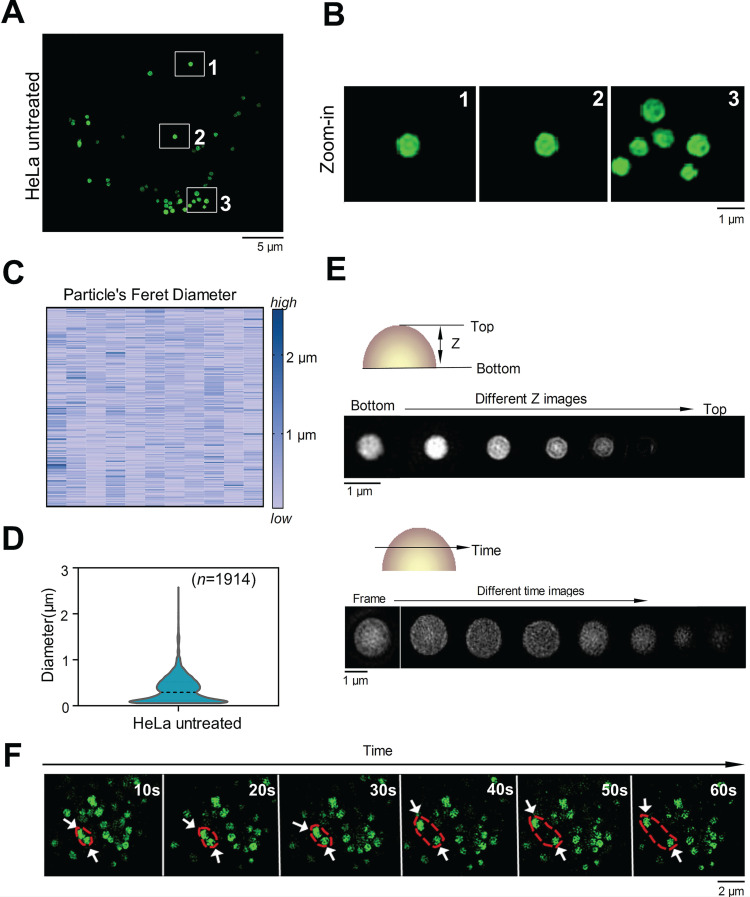
SIM images of **C-Py** puncta in HeLa cells (*λ*_ex_ = 488 nm, *λ*_em_ = 505-550 nm). (A) SIM image of cells stained with **C-Py** (10 µM) for 2 h. (B) Enlarged images of the indicated regions in A. (C and D) Size distribution of the **C-Py** puncta. Their diameters ranged from 0.1 to 3 µm (*n* = 1914). (E) Depth- and time-dependent images of cells stained with **C-Py**. (F) Images of the dynamic **C-Py** puncta over time. Two interacting puncta are outlined by the red dashed line.

To confirm whether **C-Py** targets organelles in living cells, HeLa cells were incubated with **C-Py** and SIM images were shown in **Figure 2A-B**, intracellular fluorescence from **C-Py** presented as round puncta, all of which were randomly distributed and unconnected. The diameters of these puncta ranged from 0.1 to 3.0 μm (**Figure 2C-D**) with an average diameter of 1.0 μm, which is consistent with the previously reported diameters of LDs (from 0.1 to 100 μm in different cell types) 32. To better identify these puncta, we used 3D-SIM to capture images at different depths in the cells and over time. The size and brightness of the puncta varied with depth (**Figure 2E**), which is consistent with the characteristics of LDs. Further, the dynamic processes of the puncta were observed over time, and two puncta were shown to establish contact with each other and then separate (**Figure 2F**). To assess the SIM imaging characteristics of **C-Py** in different cell lines, HepG2 cells and A549 cells were stained with **C-Py**. The images of these cells showed the same bright, round puncta (**Figure S8**).

Next, the cytotoxicity of **C-Py** to HeLa cells was evaluated using the CCK-8 assay 33. As shown in **Figure S9**, **C-Py** in the concentration range of 0 to 50 μM exerted negligible cytotoxicity to HeLa cells over 12 h, suggesting that **C-Py** is potentially safe at a working concentration of 10 μM for long-term SIM of dynamic processes in living cells. Additionally, at low concentrations of 0.1-1.0 μM, the fluorescence intensity of **C-Py** was markedly low, while at concentrations of 2.5-50 μM, **C-Py** showed sufficiently bright fluorescence and efficient cellular uptake for SIM (**Figure S10**). The uptake pattern of **C-Py** in HeLa cells was then observed under different temperatures, metabolic levels, and endocytosis levels. As shown in **Figures S11-12**, after incubation with **C-Py** for 2 h at 37 °C, the cells showed evident green fluorescence that was significantly higher than that of cells incubated with **C-Py** at 4 °C, with mitochondrial inhibitors (2-deoxy-D-glucose and oligomycin), or with an endocytosis inhibitor (NH_4_Cl) 34. This result indicates that the biosensor entered the cells through energy-dependent endocytosis. Taking together the morphology, size, distribution, and movements of the puncta, we concluded that **C-Py** labels an organelle in living cells with low toxicity and good cellular permeability.

### C-Py specifically labeled LDs with high signal to background ratio (SBR) and photostability

**Figure 3:**
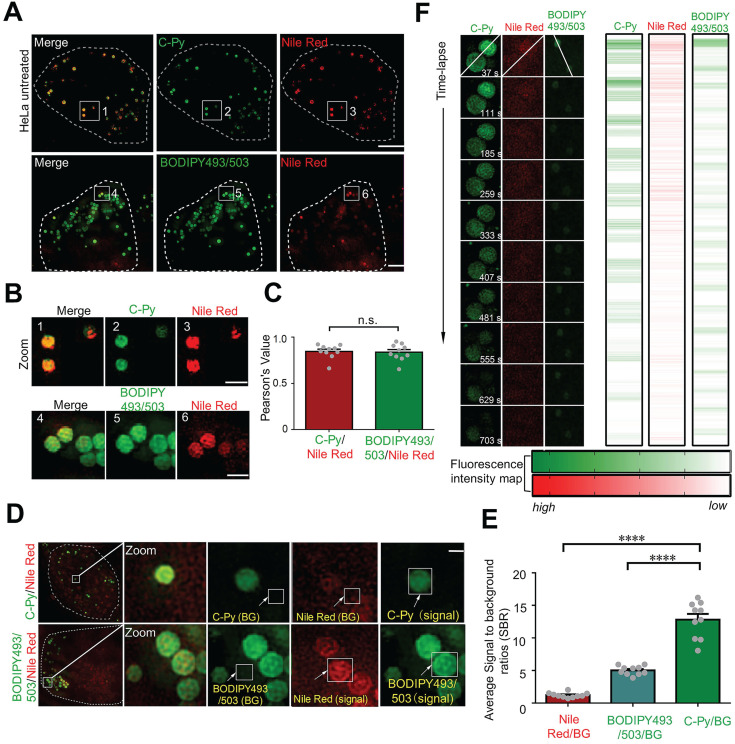
Co-localization, SBR, and photostability of **C-Py**, BODIPY493/503, and Nile Red in HeLa cells under SIM. (A) Merged SIM images of cells stained with **C-Py** or BODIPY493/503 and Nile Red. Scale bar, 5 µm. (B) Enlarged images of the indicated regions in A. Scale bar, 1 µm. (C) Quantitative analysis of the colocalization between **C-Py** or BODIPY493/503 and Nile Red. (D) Merged SIM images of cells stained with **C-Py** or BODIPY493/503 and Nile Red, with signal and background (BG) regions for each channel indicated. (E) SBRs of **C-Py**, BODIPY493/503, and Nile Red (****P<0.0001). (F) Photostability of **C-Py**, BODIPY493/503, and Nile Red during continuous irradiation with the SIM laser. Scale bar, A, 5 µm. B, 1 µm, D, 1 µm.

In cellular imaging using SIM, one of the major concerns is the specificity of the fluorescent biosensor 35. The size (0.1 to 3.0 μm) and spherical morphology of the **C-Py** puncta (**Figure 2C-D**) prompted us to further investigate whether **C-Py** specifically labels LDs. The specificity of **C-Py** was compared to that of the commercial probe BODIPY493/503 using a colocalization experiment in HeLa cells with the LD stain Nile Red. The merged images revealed that the green fluorescence signal of **C-Py** overlapped well with the red fluorescence signal of Nile Red (**Figure 3A-B**). The Pearson colocalization coefficient (*PCC*) was calculated to be as high as 0.85 for **C-Py** and Nile Red. There was no significant difference between BODIPY493/503 and Nile Red (*PCC* = 0.84), indicating the high LD labeling specificity of **C-Py** (**Figure 3C**).

A low background fluorescence is a necessary feature of an effective biosensor to avoid interference from external factors in image analysis 36. The SBRs of **C-Py**, BODIPY493/503, and Nile Red were calculated to further compare their imaging capabilities. As shown in **Figure 3D**, the green fluorescence signals of **C-Py** and BODIPY493/503 were clear and bright, while the red fluorescence signal from Nile Red was diffuse and blurry throughout the cell and had a lower intensity. The SBRs of the biosensors were markedly different (**Figure 3E**), with **C-Py** providing a lower background signal than BODIPY493/503 and Nile Red. This provided a basis for the application of **C-Py** to explore the behavior of LDs using extended-resolution imaging.

The high-power lasers used in SIM may cause photobleaching of the fluorophore. Therefore, it is extremely important that the biosensor exhibits high resistance to photobleaching to enable longitudinal imaging 37. To study the photostability of **C-Py** in living cells, continuous laser irradiation was applied over an extended period. After 703 s of continuous irradiation, the fluorescence intensity loss of **C-Py** was less than that of Nile Red and BODIPY493/503 (**Figure 3F**), which suggests that **C-Py** is resistant to photobleaching and suitable for long-term tracking of the dynamic movements of LDs in living cells by SIM.

### SIM tracking of contact sites between C-Py-labeled LDs and multicellular organelles

**Figure 4:**
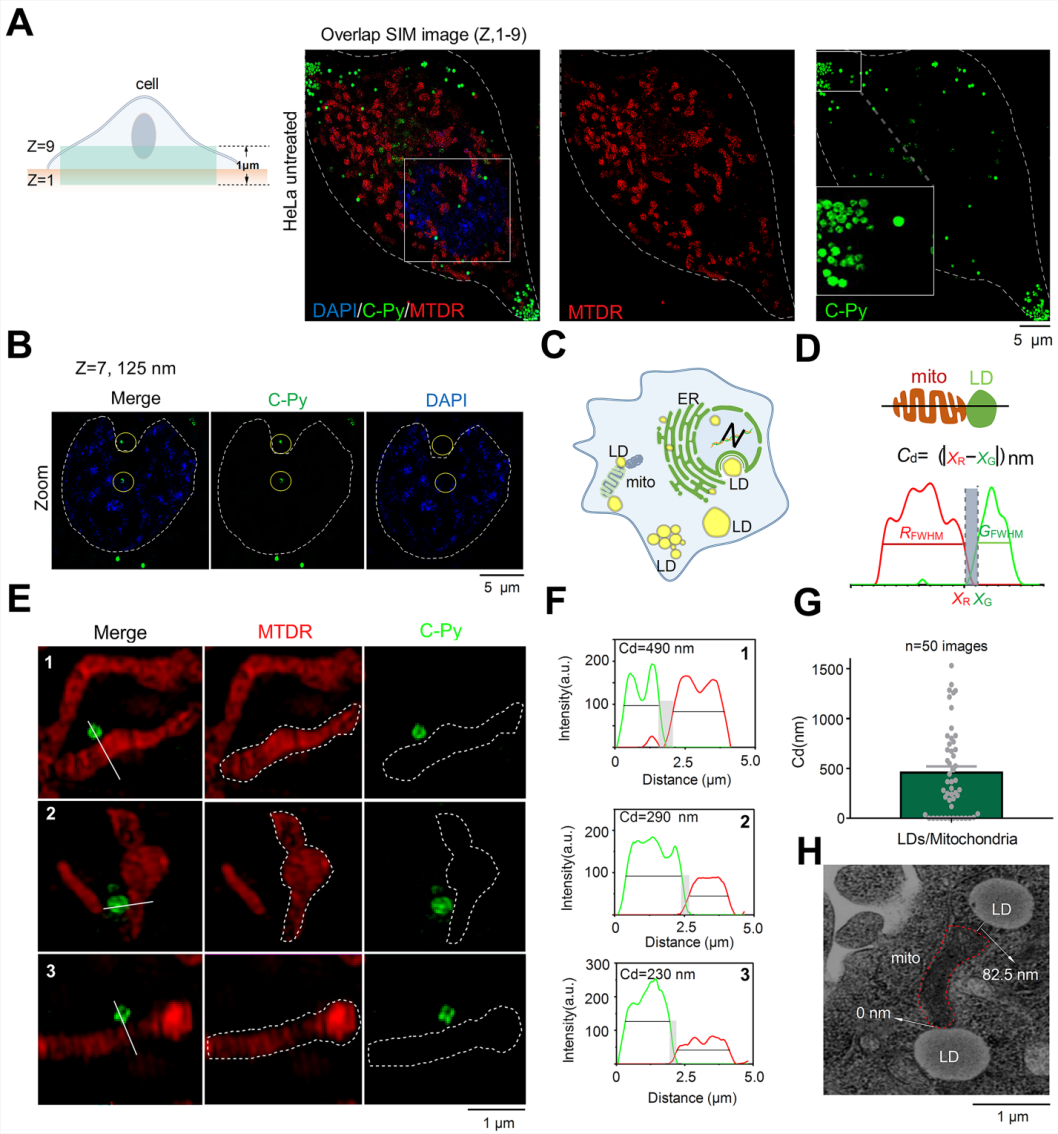
**C-Py** nanoscopic tracking of organelle-LD contact sites in living cells. (A) 3D-SIM image collected over 1 μm in the Z-axis of LDs (**C-Py**), mitochondria (MTDR), and nuclei (DAPI). (B) SIM images collected at 125 nm in the Z-axis of LDs and nuclei. (C) Schematic diagram of the distribution of LDs and other organelles. (D) Schematic diagram of the metric used for estimating the contact distance between LDs and mitochondria. *C*_d_ is the absolute distance between the edge of a red mitochondrion (*x*_R_) and a green LD (*x*_G_) on the x-axis, which are derived from the FWHM of the organelle images. (E) SIM images of contact sites between LDs and mitochondria. (F) Fluorescence intensity profiles along the solid white lines in E and the calculated *C*_d_ values. (G) LD-mitochondria contact distances measured using SIM. Data are represented as mean ± SEM (*n* = 50 images from 20 cells) and three independent experiments were analyzed. (H) TEM image of contact sites between a mitochondrion and LDs.

LDs not only store energy but also establish close interactions with other organelles that are crucial for the regulation of cell membrane transport, lipid metabolism, protein degradation, and signal transduction 38-40. To explore the interactions between LDs and other organelles, cells were co-stained with **C-Py**, 4',6-diamidino-2-phenylindole (DAPI), and Mito Tracker Deep Red (MTDR) to label LDs, nuclei, and mitochondria, respectively. Because organelles in whole cells have a certain thickness, it is difficult to obtain a clear panorama using conventional optical microscopy. Therefore, samples were imaged at multiple layers with 125 nm per frame by 3D-SIM to reduce false positives 41, 42 (**Figure S13**). As shown in **Figure 4A**, 9 image layers were captured in the Z-axis direction to a depth of 1 μm to show the distribution of LDs in various parts of the cell and close contacts with mitochondria and nuclei. As shown in **Figure 4B**, in the 7^th^ layer of the 3D image at a Z-axis depth of 125 nm, LDs were found in the nuclear region. As shown in **Figure S14A**, LDs and nuclei could be clearly seen in layers 5, 7 and 9, and LDs could be clearly seen in nucleus in **Figure S14B**, further confirming that LDs were located in the nucleus. LDs are considered to be confined to the cytoplasm, even though it has been reported that there are relatively substantial amounts of LDs present in the nucleus in certain cell lines (**Figure 4C**) 43. Herein, the nuclear distribution of LDs in HeLa cells was revealed by extended-resolution imaging using **C-Py**.

LDs provide energy to mitochondria by forming contact points. Damage to the energy supply process, or contact site, results in the development of disordered lipid metabolism and impaired mitochondrial function 44-47. The resolution of conventional microscopes is insufficient to clearly observe organelles in living cells, which markedly restricts our ability to image contact sites between LDs and mitochondria and to understand related metabolic diseases. To solve this issue, we used **C-Py** to track contact sites between LDs and mitochondria in living cells by SIM. First, we proposed the metric *C*_d_ = |*x*_R_ - *x*_F_| (nm) for quantification of the contact distance between mitochondria and LDs (**Figure 4D**). This metric uses the full width at half maximum (FWHM) of the fluorescence signal to define the edges of the organelles, which reflects the resolution of the image. Using this metric, mitochondria and LDs were observed to be in close contact, forming contact sites shorter than ~450 nm under normal physiological conditions (**Figure 4E-G**). In addition, we captured continuous dynamic images of LDs and mitochondria from separation state to contact point by SIM. Results as shown in the**Figure S15**, LDs and mitochondria were in a non-contact state at 31s. With the change of time, LDs and mitochondria were partially contact at 186 s. The results showed that they could establish a specific contact point. Then, we imaged contact sites between LDs and mitochondria by transmission electron microscopy (TEM), and found them to be shorter than 100 nm on average (**Figure 4H**). We also used **C-Py** and Dil to track the LDs and membrane in living cells and the results showed that there were a lot of LDs near the cell membrane (**Figure S16**) 41. However, the current quantitative analysis mainly relies on manual, an automatic analysis tool that can quantify the distance between LDs and mitochondria is lacking, which requires the help of experts in computer image recognition. We believe that in the future, the development of image algorithms and artificial intelligence (AI) will provide convenience for researchers to analyze images for organelle interaction.

In summary, interactions between LDs and multiple organelles, especially LD-mitochondria contact sites, were imaged at the nanoscale using **C-Py**. These results provide a new starting point for investigating the role and regulation of LDs in living cells at the nanoscale.

### LPS decreases the number of contact sites between LDs and mitochondria

Apart from being a storage site of neutral lipids, LDs have been regarded as the basic platform for the execution of various cellular processes, including immune responses. Arachidonic acid-like enzymes, which are important signaling molecules involved in inflammatory responses, including cyclooxygenase, prostaglandin E_2_ synthetase, and leukotriene C4 synthetase, also exist in LDs 48. When host cells are infected by pathogenic microorganisms, LDs redistribute and produce the required inflammatory mediators to regulate the immune response 48. Further, research has also suggested that mitochondria are the key centers of the elicitation of innate immune responses to antibiotics and antiviral agents 10, 49, 50. However, interactions between LDs and mitochondria in inflammatory responses have rarely been studied 10, which hinders the precise evaluation of the mechanisms underlying organelle interactions under states of inflammation and the subsequent discovery of anti-inflammatory drugs.

**Figure 5:**
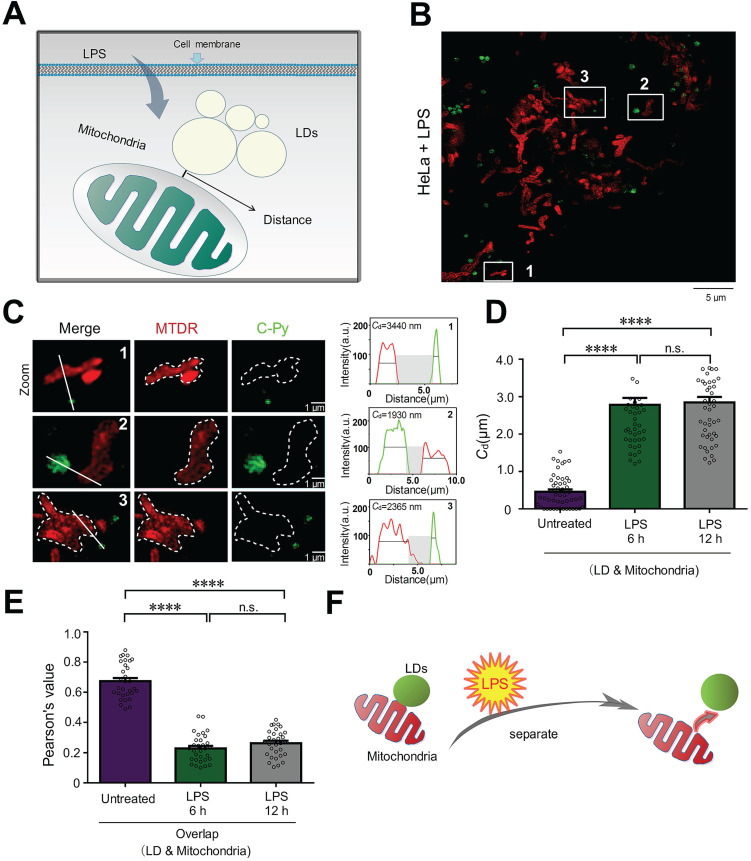
LPS decreases the number of LD-mitochondria contact sites. (A) Schematic diagram illustrating the predicted changes to the mitochondrial contact sites of LDs during inflammatory processes. (B) SIM image of contact sites established between LDs (**C-Py**) and mitochondria (MTDR) after induction by LPS. (C) Enlarged images of the indicated regions in B, and quantitative analysis of the fluorescence intensity profiles along the solid white lines. (D) Contact distances between LDs and mitochondria in living cells without LPS stimulation or with LPS stimulation for 6 or 12 h. Data are represented as mean ± SEM (*n* = 51 from 10 images). Statistical differences between two groups were examined using Mann-Whitney tests (****P < 0.0001). (E) Quantitative analysis of the colocalization between LDs and mitochondria in cells untreated or treated with LPS for 6 or 12 h. Data are represented as mean ± SEM (*n* = 31 from 31 images) and three independent experiments were analyzed. Statistical differences between two groups were examined using Mann-Whitney tests (****P < 0.0001). (F) Schematic diagram illustrating the relationship between LDs and mitochondria following LPS treatment.

To investigate the changes to contact sites between LDs and mitochondria under pathological conditions such as inflammatory processes, HeLa cells were treated with 30.0 μg/mL LPS for 6 and 12 h to induce an inflammatory reaction in the cells (**Figure 5A**). Then, LDs and mitochondria were stained with **C-Py** and MTDR, respectively. After treatment, the LPS-induced mitochondria were physically separated from the LDs. After LPS treatment for 6 h, the average contact distance between LDs and mitochondria increased from 458.4 ± 60.7 nm to 2786 ± 180.8 nm, and after 12 h, the average contact distance increased to 2847 ± 148.3 nm (**Figure 5B-D**). Moreover, the *PCC* of LDs and mitochondria decreased from 0.6737 ± 0.0211 in untreated HeLa cells to 0.2291 ± 0.0165 and 0.2633 ± 0.0159 after 6 and 12 h of LPS induction (**Figure 5E**), which verified that LDs uncoupled from mitochondria with LPS induction. When cells starve, LDs degrade triglycerides into fatty acids and provide them to the mitochondria, which then produce ATP through oxidative phosphorylation (OXPHOS) 51. It has been reported that cellular innate immune stimulation increases aerobic glycolysis and reduces OXPHOS 51. Therefore, we speculate that LPS-induced uncoupling of LDs and mitochondria may contribute to the reduction of OXPHOS and play an anti-inflammatory role. This result promotes the importance of morphological studies of interactions between intracellular organelles during inflammation and provides a theoretical basis for the development of anti-inflammatory drugs.

## Conclusion

Physical contact between LDs and other organelles, especially mitochondria, plays a key role in the intracellular transport of metabolites. Monitoring interactions between organelles in living cells by fluorescence microscopy is an effective approach to functionally evaluating these physiological processes. However, owing to the diffraction limit of light, accurate target acquisition is usually limited. Furthermore, LDs and mitochondria are highly dynamic subcellular organelles that show significant subcellular movement. It is highly desirable to image these organelles and their interactions with increased resolution and higher speed. Therefore, a LD-targeted biosensor, **C-Py**, with excellent photostability, low cytotoxicity, high cellular permeability, and good photostability was developed for imaging the cellular dynamics of LDs and their interactions with multiple organelles by SIM. **C-Py** was successfully used to observe the contact sites established between LDs and mitochondria at the nanoscale in living cells, and revealed that the number of contact sites is decreased under LPS-induced inflammation. Our probe also revealed LDs located the nucleus area and LDs interact with mitochondria in living cells under SIM, instead of traditional electron microscopy. Thus, **C-Py** not only provides strategies for the design of ultra-highly selective biosensors but may also become a powerful approach for investigating LD biology.

## Supplementary Material

Supplementary figures and scheme.

## Abbreviations

LDs: lipid droplets
STED: stimulated emission depletion
STORM: stochastic optical reconstruction microscopy
PALM: photo-activated localization microscopy
SIM: structured illumination microscopy
LMC: LDs and the mitochondria contact site
DMEM: Dulbecco's modified Eagle's medium
SBR: signal-to-background ratios
LPS: Lipopolysaccharide
MTDR: MitoTracker Deep Red
DAPI: 4',6-Diamidino-2-Phenylindole, Dihydrochloride
DiL: 1,1'-Dioctadecyl-3,3,3',3'-Tetramethylindocarbocyanine Perchlorate
CCK-8: Cell Counting Kit-8
ICT: intramolecular charge transfer
SBR: signal-to-background ratio
TEM: traditional transmission microscope
FWHM: full width at half-maximum
OXPHOS: oxidative phosphorylation
