# Supplementary material for: Long-term live-cell lipid droplet-targeted biosensor development for nanoscopic tracking of lipid droplet-mitochondria contact sites
Source: Theranostics. 2021 Jun 22;11(16):7767–78. doi: 10.7150/thno.59848 (PMC8315056; doi:10.7150/thno.59848)
Supplement: Supplementary file 1 — Supplementary figures and scheme. [file thnov11p7767s1.pdf]

Supporting Information

Figure S1. Structure of Nile Red and **C-Py**.
Scheme. S1. Synthetic route for **C-Py**.
Figure S2. HR-Mass spectrum of **C-Py**.
Figure S3. ^{1}H-NMR spectrum of **C-Py**.
Figure S4. ^{13}C-NMR spectrum of **C-Py**.
Figure S5. Bright field images of untreated HeLa cells.
Figure S6. Reconstructed SIM images (A, B and C) stained with **C-Py**, were imaged identically A was acquired with 1.516 (*n*@589.3 nm) immersion oil and B, C with 1.524,1.510 (*n*@589.3 nm) immersion oil.
Figure S7. Reconstructed SIM images (A, B and C) stained with **C-Py**, were imaged identically A was acquired with 1.516 (*n*@589.3 nm) immersion oil and B, C with 1.524,1.510 (*n*@589.3 nm) immersion oil.
Figure S8. **C-Py** tracking LDs in HepG2 cells and A549 cells under SIM.
Figure S9. Cytotoxicity of the **C-Py** at concentrations of 0.1-50 μmol/L in HeLa cells.
Figure S10. Permeability of the **C-Py** at concentrations of 0.1-50 μmol/L in HeLa cells.
Figure S11. SIM images of HeLa cells of **C-Py** under different conditions.
Figure S12. Normalized fluorescence intensity of **C-Py** in HeLa cells under different stimulations.
Figure. S13. Optical resolution in z of 3D-SIM, Epi-illumination fluorescence microscopy and confocal mroscopy.
Figure. S14. **C-Py** nanoscopic tracking of the nucleus–LDs interaction in HeLa cells.
Figure. S15. The formation of contact sites between LDs (**C-Py**) and mitochondria (MTDR).
Figure S16. **C-Py** and Dil tracking LDs and membranes in HeLa cells under SIM.

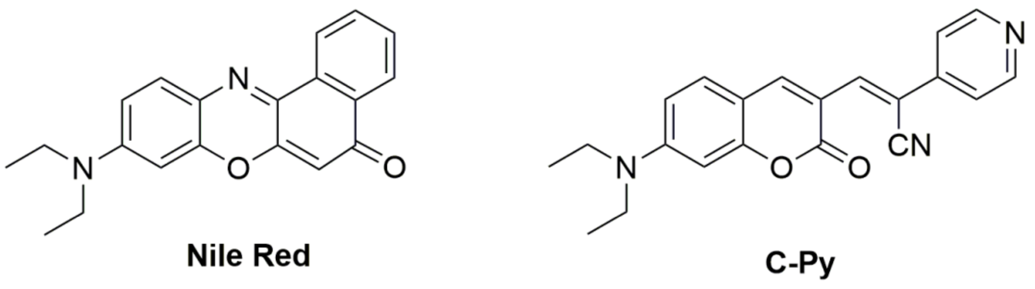

Figure S1. Structure of Nile Red and **C-Py**.

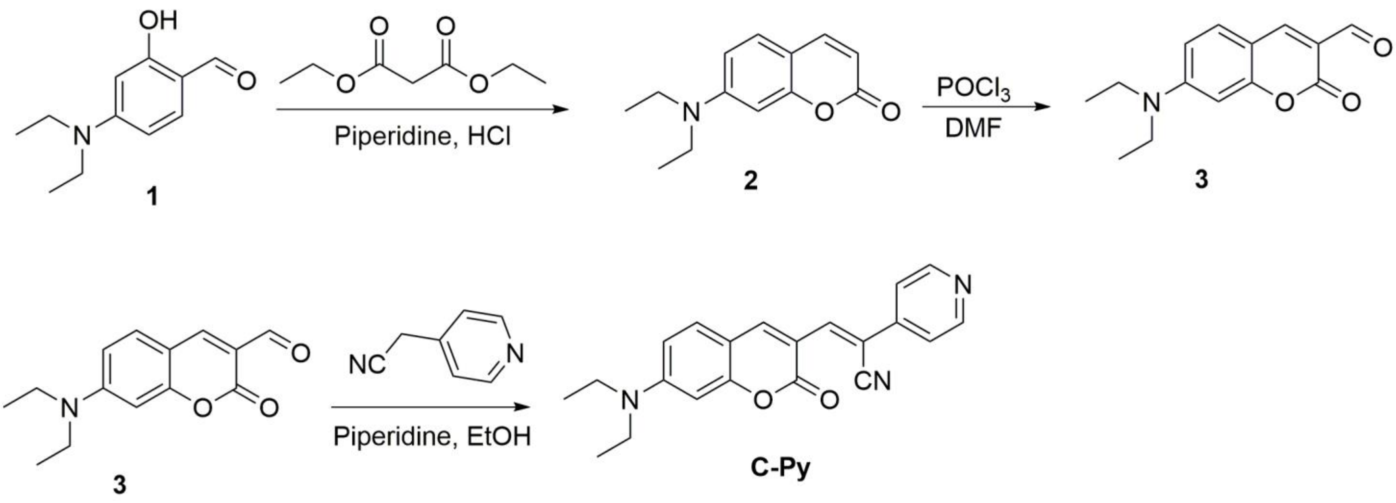

Scheme. S1. Synthetic route for **C-Py**.

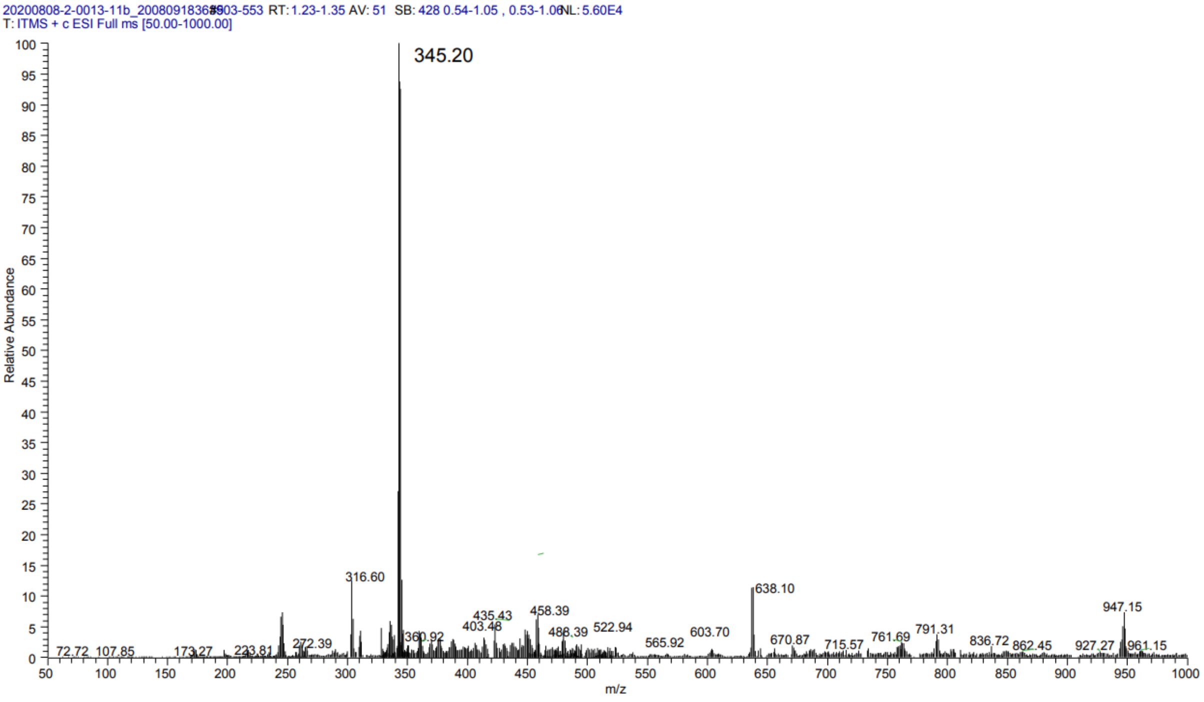

Figure. S2. HR-Mass spectrum of **C-Py**.

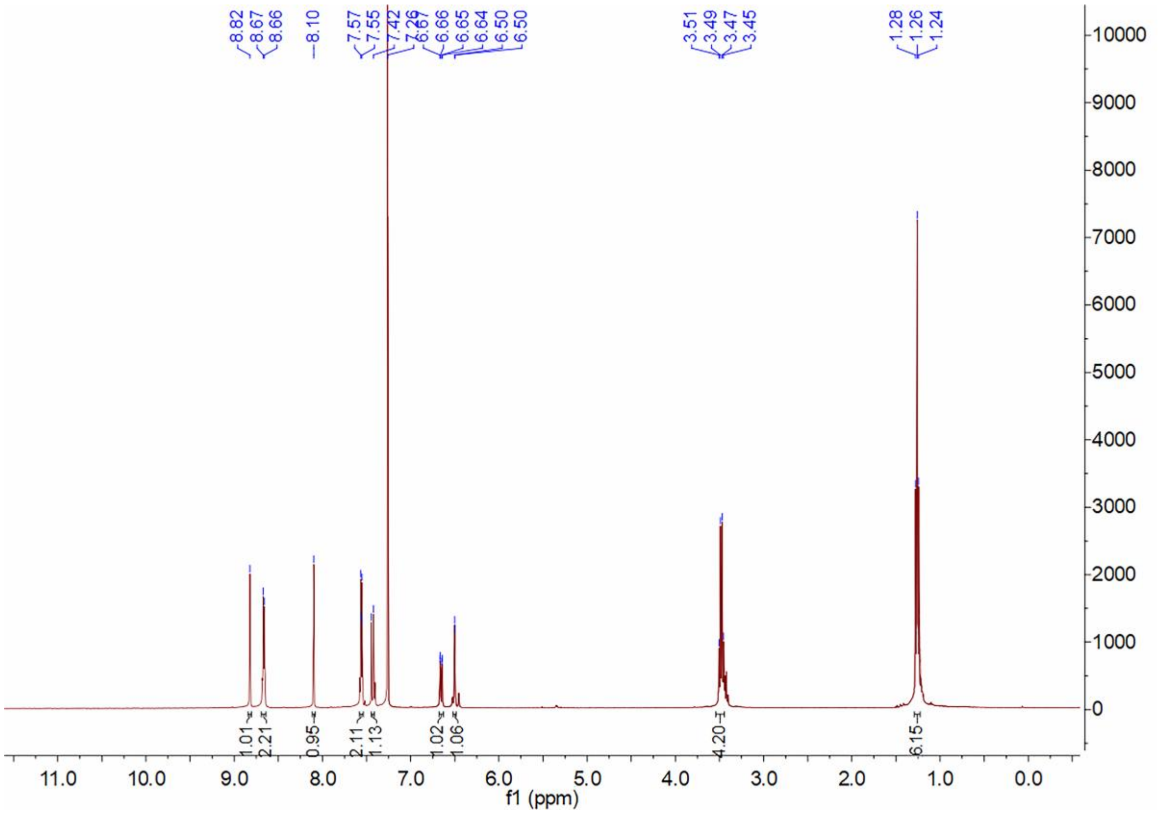

Figure. S3. ^{1}H-NMR spectrum of **C-Py**.

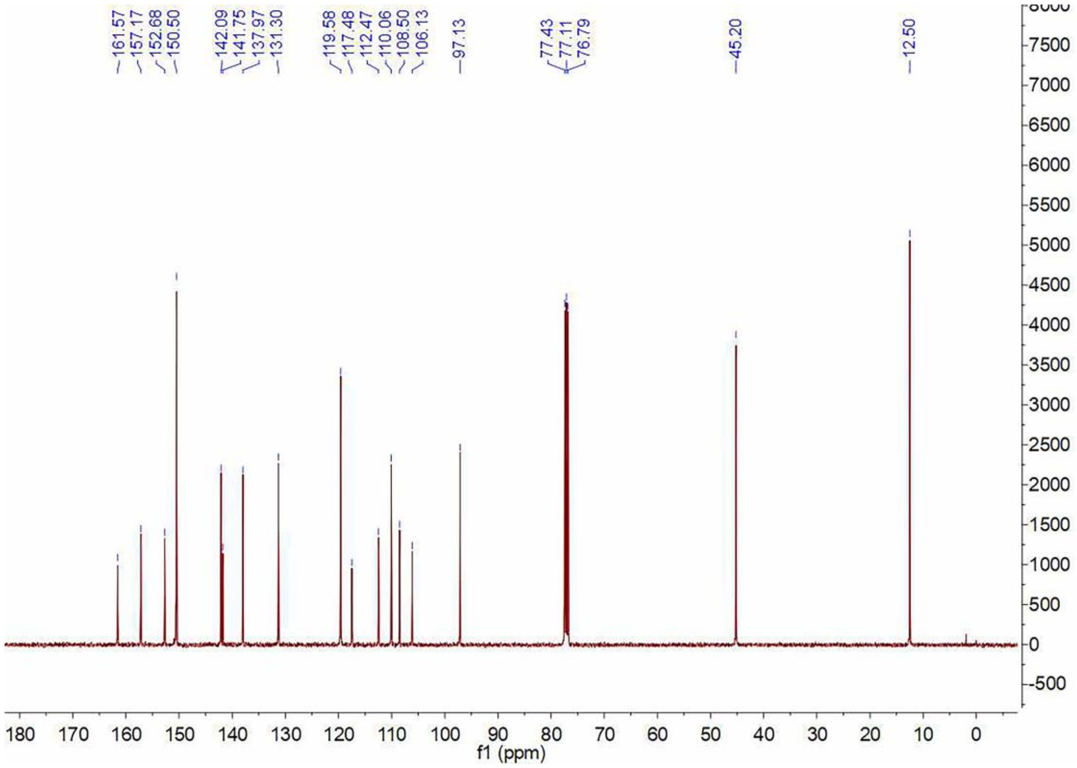

Figure. S4. ^{13}C-NMR spectrum of **C-Py**.

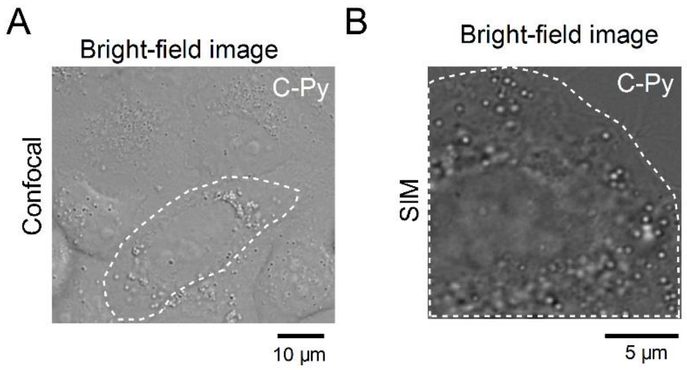

Figure S5. Bright field images of untreated HeLa cells. (A) Bright field image of **C-Py** under Confocal. (B) Bright field image of **C-Py** under SIM.

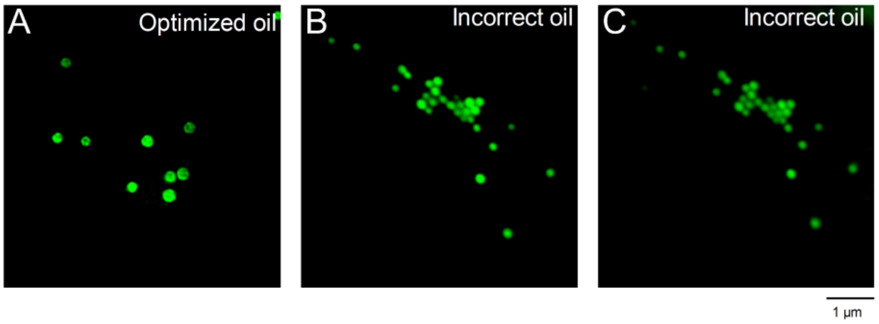

Figure S6. Reconstructed SIM images (A, B and C) stained with **C-Py**, were imaged identically A was acquired with 1.516 (*n*@589.3 nm) immersion oil and B, C with 1.524,1.510 (*n*@589.3 nm) immersion oil.

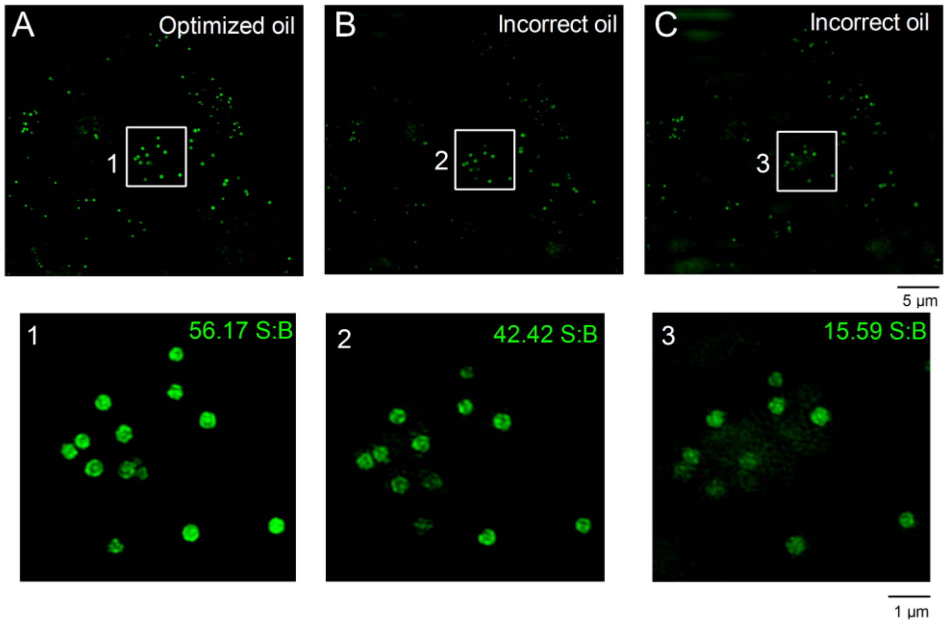

Figure S7. Reconstructed SIM images (A, B and C) stained with **C-Py**, were imaged identically A was acquired with 1.516 (*n*@589.3 nm) immersion oil and B, C with 1.524,1.510 (*n*@589.3 nm) immersion oil.

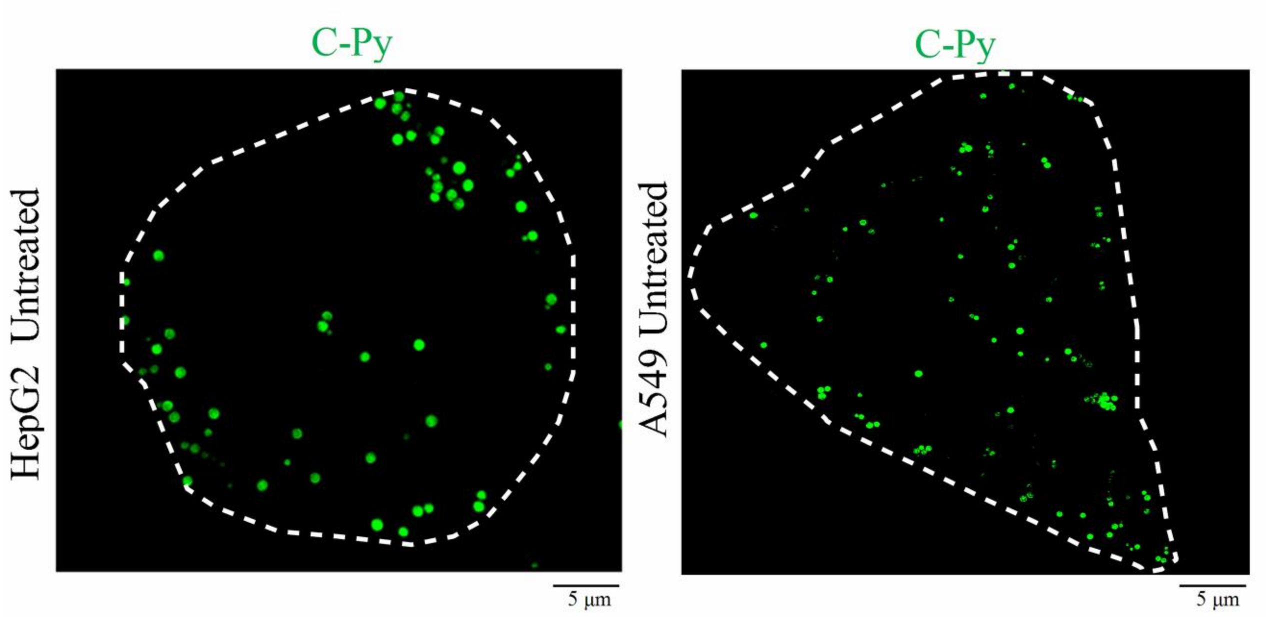

Figure. S8. C-Py tracking LDs in HepG2 cells and A549 cells under SIM.

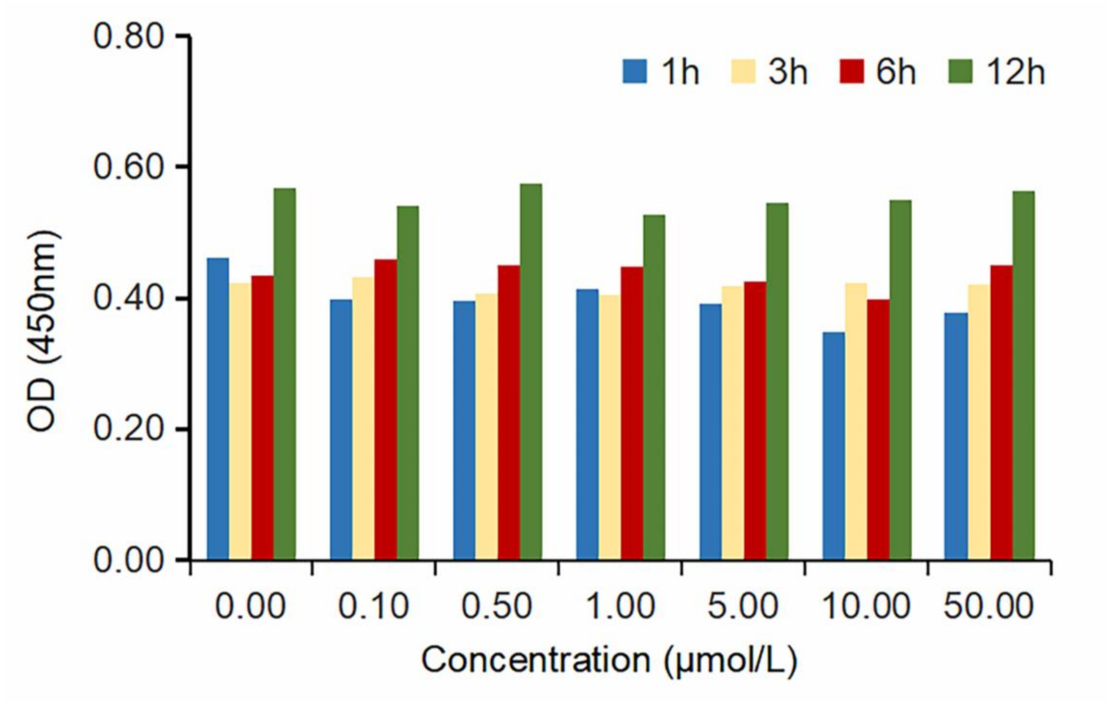

Figure. S9. Cytotoxicity of the **C-Py** at concentrations of 0.1-50 μmol/L in HeLa cells.

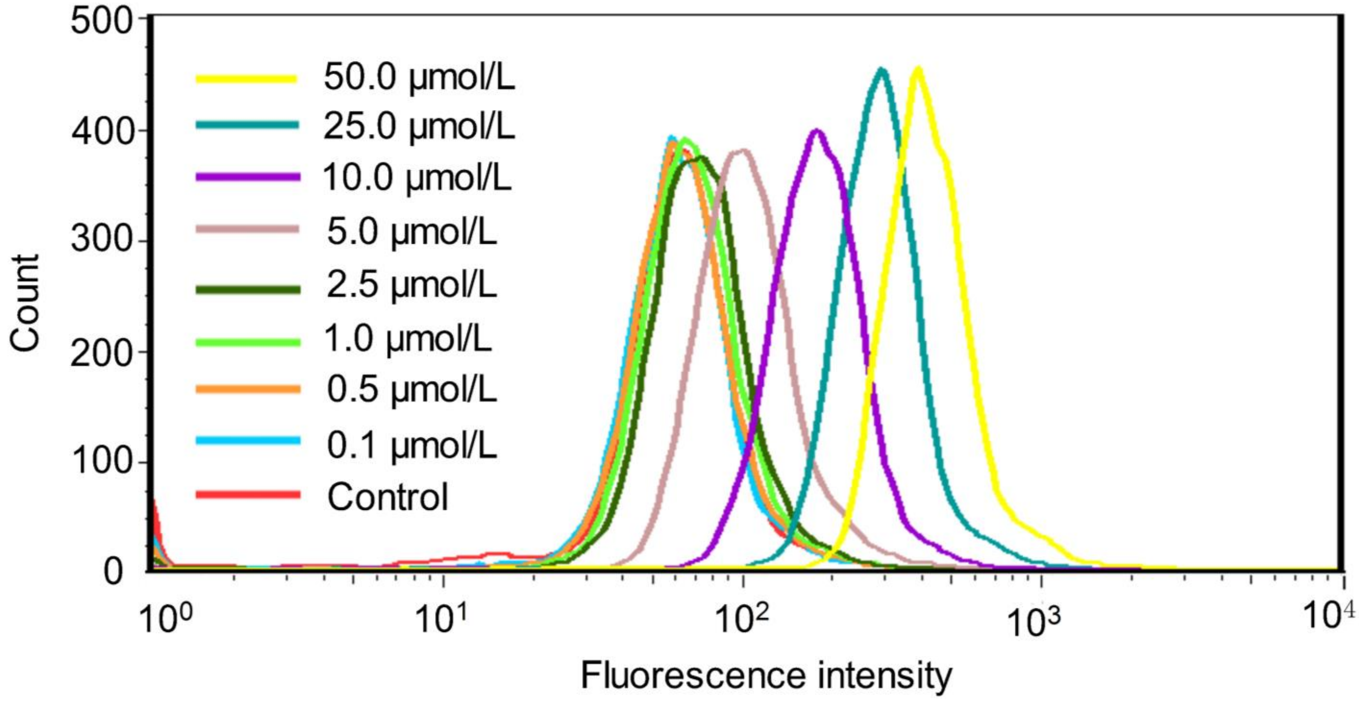

Figure. S10. Permeability of the **C-Py** at concentrations of 0.1-50 μmol/L in HeLa cells.

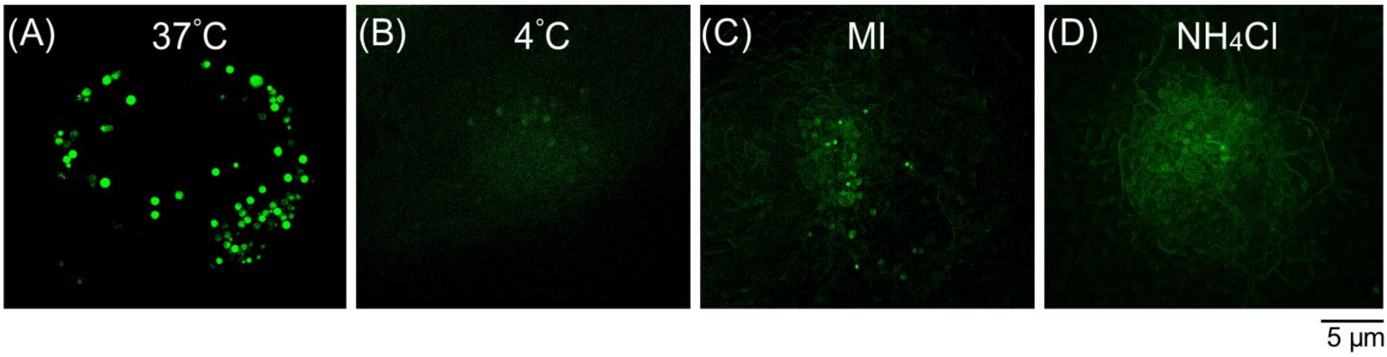

Figure. S11. SIM images of HeLa cells of **C-Py** under different conditions. (A) HeLa cells were incubated with **C-Py** for 2h at 37 °C; (B) HeLa cells were incubated with **C-Py** for 2h at 4 °C. (C) HeLa cells were incubated with the metabolic inhibitors (MI, including 50 mM oligomycin and 5 μM 2-deoxy-D-glucose) at 37 °C for 1h and incubated with **C-Py** at 37 °C for 2h. (D) HeLa cells were incubated with 50 mM NH_4Cl at 37 °C for 1 h and with **C-Py** at 37 °C for 2h.

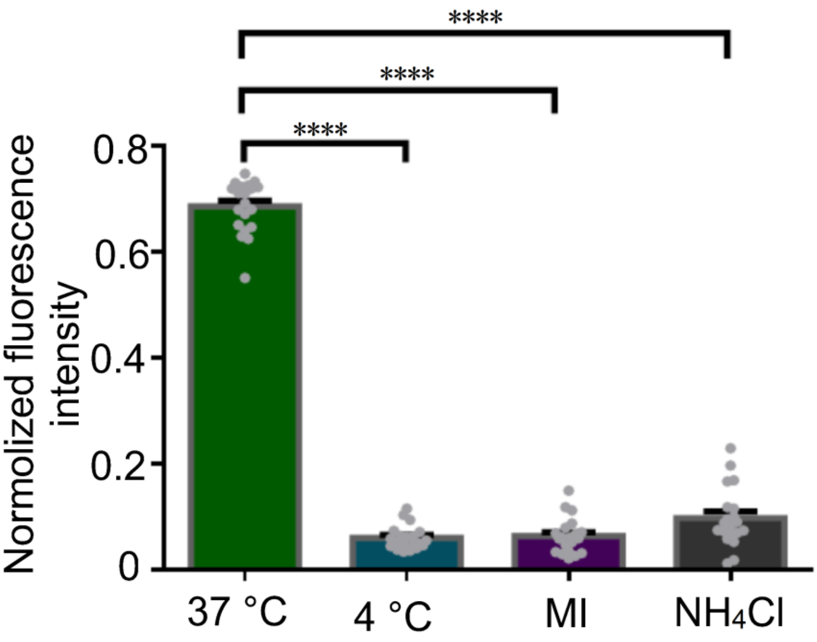

Figure. S12. Normalized fluorescence intensity of **C-Py** in HeLa cells under different stimulations. Data are presented as mean ± SEM (n =20, $^{****}P < 0.0001$).

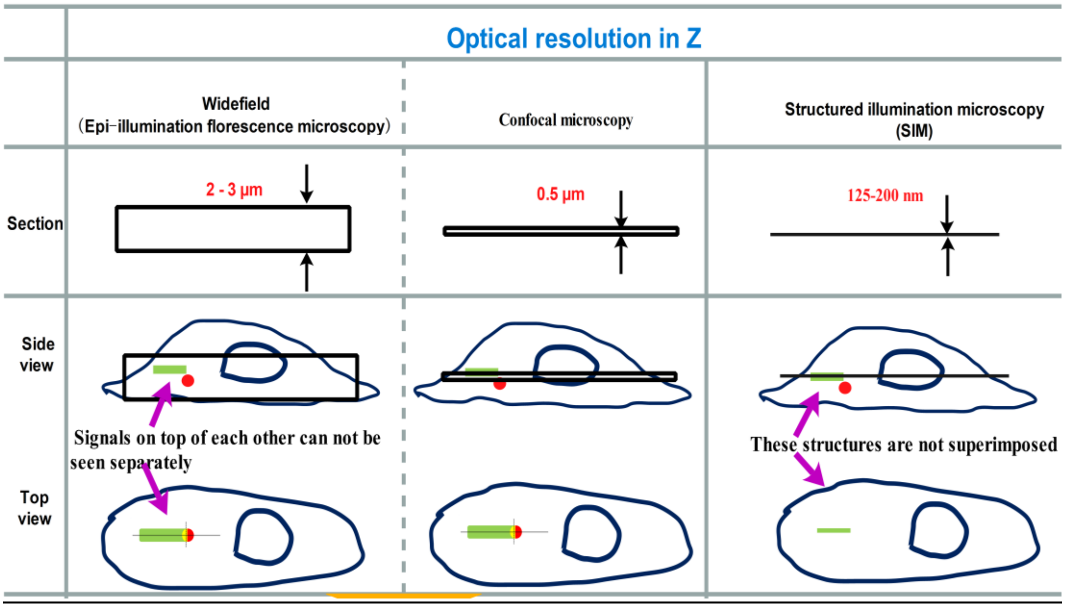

Figure. S13. Optical resolution in Z of 3D-SIM, Epi-illumination fluorescence microscopy and confocal mroscopy.

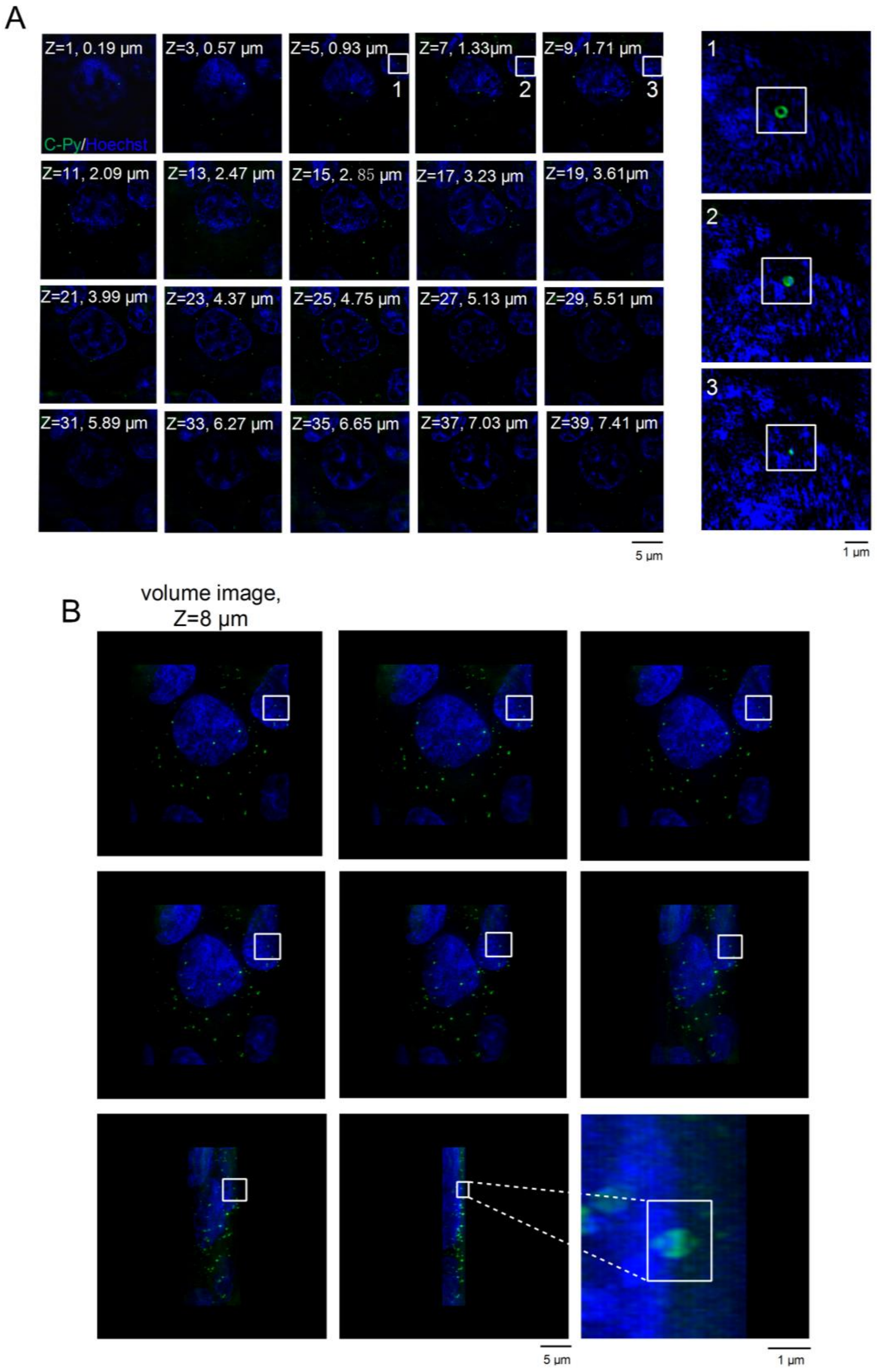

Figure S14. C-Py nanoscopic tracking of the nucleus–LDs interaction in HeLa cells. SIM images at 8 μm at the Z axis of the LDs (**C-Py**) and nucleus (Hoechst 33342). A. z1-39 layers of SIM image, B. 3D SIM images from different angles. The solid white frame represents the LDs in the nucleus.

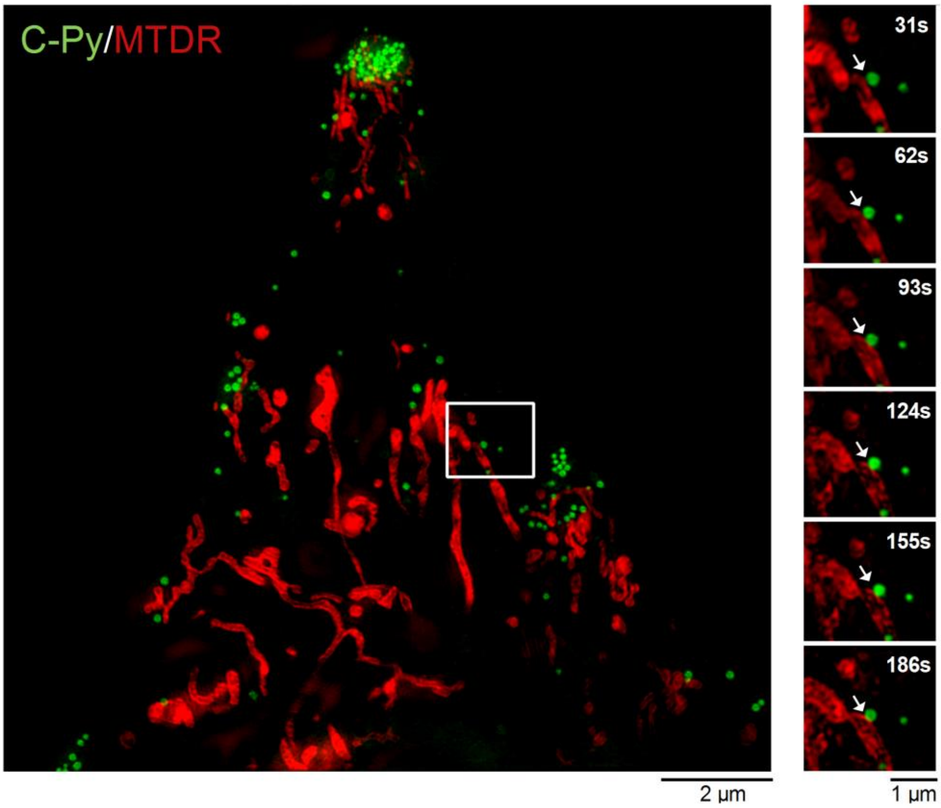

Figure. S15. The continuous dynamic image of LDs (C-Py) and mitochondria (MTDR) under SIM

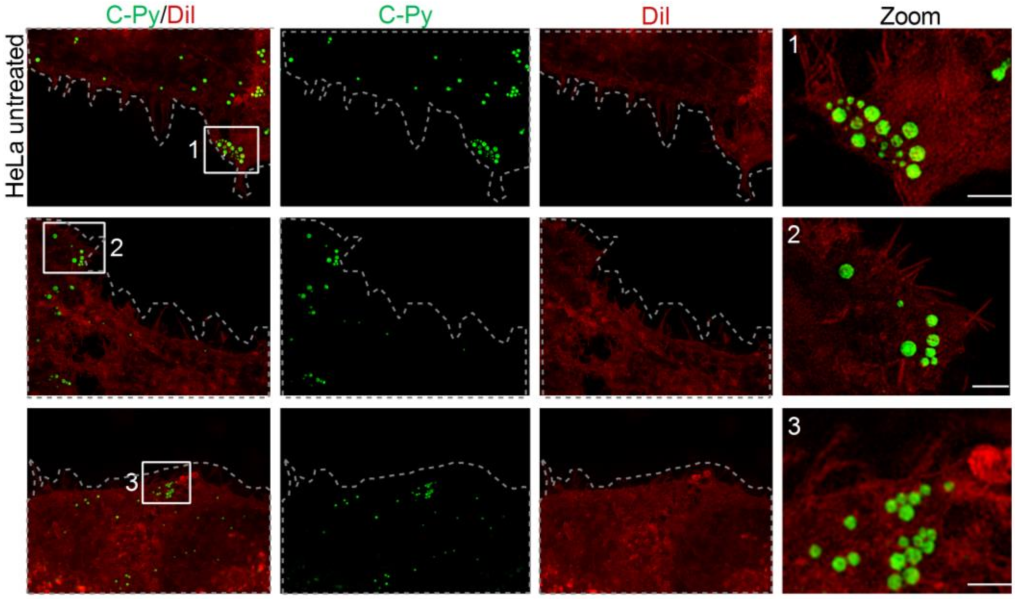

Figure. S16. Using **C-Py** and Dil track LD and membranes, respectively, in HeLa cells under SIM. The white dotted line refers to the cell membrane. Scale bar, 1μm.
